# Association Between Patient Characteristics and the Depth of Microvascular Penetration Into the Adult Human Meniscus

**DOI:** 10.1177/03635465241307216

**Published:** 2025-01-27

**Authors:** Thies J.N. van der Lelij, Peter van Schie, Amber Weekhout, Marta Fiocco, Roelina Munnik-Hagewoud, Stijn Keereweer, Hans Marten Hazelbag, Ewoud R.A. van Arkel, Pieter B.A.A. van Driel

**Affiliations:** † Department of Orthopaedics, Leiden University Medical Center, Leiden, The Netherlands; ‡ Mathematical Institute Leiden University, Leiden, The Netherlands; § Department of Biomedical Data Science, Leiden University Medical Centre, Leiden, The Netherlands; ‖ Department of Orthopaedics, Isala Hospital, Zwolle, The Netherlands; ¶ Department of Otorhinolaryngology, Erasmus Medical Center, Rotterdam, the Netherlands; # Department of Pathology, Haaglanden Medical Center, The Hague, The Netherlands; ** Department of Orthopaedics, Haaglanden Medical Center, The Hague, The Netherlands; Investigation performed at Haaglanden Medical Centre, The Hague, The Netherlands

**Keywords:** meniscus, meniscal tear, meniscal healing, vascularization, blood supply

## Abstract

**Background:**

Current knowledge on the microvascular anatomy of adult human menisci is based on cadaveric studies. However, considerable interindividual variation in meniscal microvascularization has been reported in recent studies with small sample sizes.

**Purpose:**

To assess the association between patient characteristics and the depth of microvascularization of the meniscus.

**Study Design:**

Descriptive laboratory study.

**Methods:**

Menisci from 174 patients who received total knee replacement between March 2021 and December 2023 were collected. A total of 174 lateral and 102 medial menisci were included. Three sections were made from each meniscus: the anterior horn, midbody, and posterior horn. Immunohistochemical staining (CD-31) was used to visualize the microvasculature. The 4 primary outcome measures were the correlation between the depth of microvascular penetration into the lateral meniscus (0%-100%) and (1) age, (2) smoking, (3) degree of osteoarthritis, and (4) history of cardiovascular disease. To account for repeated measurements within each patient, a linear mixed-effects model was estimated to study the association between microvascularization and the patient’s characteristics previously introduced.

**Results:**

The depth of vascular penetration ranged from 0% to 78% into the lateral menisci and from 0% to 67% into the medial menisci. No significant correlations were found between age, smoking, degree of osteoarthritis, or history of cardiovascular disease and the degree of vascular penetration into the lateral meniscus. The linear mixed-effects model analysis, adjusted for location within the meniscus (anterior horn, midbody, or posterior horn) and meniscal side (lateral or medial), showed no significant associations between the same patient characteristics and meniscal microvascularization.

**Conclusion:**

The degree of microvascular penetration into the meniscus has a wide range among adults >50 years. However, the depth of microvascularization was not associated with age, smoking, degree of osteoarthritis, or history of cardiovascular disease.

**Clinical Relevance:**

Because no associations were found between depth of microvascular penetration into the meniscus and patient characteristics, the latter cannot be used to estimate the vascular status of a meniscal tear in clinical practice.

In cases of a meniscal tear resulting in pain or dysfunction, meniscal repair is often preferred over meniscectomy, as (partial) meniscectomy predisposes the knee to an increased risk of osteoarthritis in the long term.^21^ However, a large proportion of meniscal tears are considered irreparable, and despite advances in arthroscopic techniques, modern meniscal repair has an overall failure rate of around 20%.^18,24^ It has been shown that the success of meniscal repair depends on the anatomic location of the tear, which is thought to be due to the differences in vascular supply.^11,12^

Identifying the presence of an association between patient characteristics and the extent of meniscal vascularization may contribute to a more accurate estimation of the microvascular anatomy of the meniscus of individual patients. Subsequently, in the case of a meniscal tear, this could help to estimate the vascular status of a tear and determine its healing potential. Meniscal repair may be the preferred treatment when a tear is considered well vascularized, whereas (partial) meniscectomy may be preferred if there is no expected adequate blood supply at the site of a tear. An individualized treatment approach for meniscal tears, based on a patients’ microvascular anatomic characteristics, may contribute to lower failure rates of meniscal repair.

The meniscus is entirely vascularized at birth, and its microvascular supply decreases from the inner to the peripheral margin during childhood.^10,25^ In the human adult meniscus, only the outer third (“red-red” zone) is considered well-vascularized.^2,5^ Importantly, large interindividual variation in vascular penetration patterns of the meniscus of adults has recently been described.^7,33^ Whether vascularization further decreases with increasing age in adults has remained subject to debate.^2,7,22,32^ Next to age, other patient characteristics, such as the degree of osteoarthritis of the knee joint, have been suggested to affect the microvascularization of the meniscus.^3,32^

Risk factors such as hypertension, hypercholesterolemia, diabetes mellitus, and obesity that alter the structure and function of microscopic blood vessels have been associated with cardiovascular disease (CVD) (ie, coronary heart disease, stroke or transient ischemic attack, and peripheral arterial disease).^13,14^ Microvascular diseases, such as retinopathy and nephropathy, are often coprevalent with CVD due to shared risk factors.^15,20,23^ Therefore, a history of CVD may be associated with the degree of microvascularization of the meniscus. Last, smoking is a risk factor that has been associated with vessel wall injury and capillary loss.^19,28^

The purpose of this study was to assess the presence of possible associations between patient characteristics and depth of microvascular penetration into the human adult meniscus. We hypothesized that the depth of microvascular penetration diminishes with increasing age, smoking, higher degree of osteoarthritis, and a history of CVD. The secondary goal was to study the extent of variation in microvascularization of the meniscus in a large group of adult patients.

## Methods

### Patients

Menisci were retrieved from patients who received total knee replacement (TKR) because of osteoarthritis. All surgeries were performed in 2 large peripheral hospitals (Haaglanden Medical Center, The Hague, The Netherlands, and Isala hospital, Zwolle, The Netherlands) between March 2021 and December 2023. Patients with previous meniscal surgery or a history of meniscal tears were excluded. Patient characteristics and clinical data were retrieved from the electronic patient file. After pseudonymization, data were collected using Castor EDC (Castor Electronic Data Capture). The study key for pseudonymization was stored on a secured disk at the local sites. The study protocol was presented to the Medical Ethics Committee Leiden The Hague Delft, who waived the need for approval under Dutch law (B20.043) as it did not concern research where participants are subjected to procedures or are required to follow rules of behavior. All patients included in the study gave their written informed consent for the use of their menisci and pseudonymized data.

### Specimen Preparation

**Figure 1.**
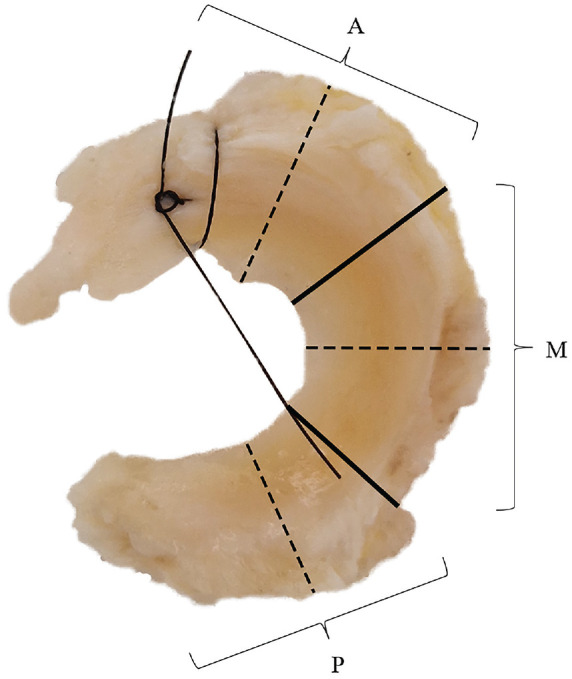
Meniscal specimen after retrieval with a suture through the anterior horn. The menisci were divided into 3 anatomic radial locations (anterior horn, midbody, and posterior horn) as shown with the solid lines. The dashed lines indicate where the tissue was transected to produce the histological sections. A, anterior horn; M, midbody; P, posterior horn.

Retrieval of menisci was performed during TKR. The lateral meniscus was chosen for the primary outcomes of this study, as the capsular attachment to the meniscal specimens was needed for the adequate measurement of microvascular penetration, which can be challenging in the medial meniscus due to its close connection with the medial collateral ligament (which should be preserved during TKR). However, if deemed possible by the operating surgeon, the medial meniscus was collected as well. After the arthrotomy was performed, the lateral and medial menisci, including a rim of capsular tissue, were removed. After retrieval, the anterior horns of the menisci were marked with a suture for orientation during histological examination. All menisci were pseudonymized and stored in formaldehyde before they were taken to the pathology department of the Haaglanden Medical Center (The Hague, The Netherlands), where specimens were divided into 3 anatomic radial locations and embedded in paraffin (Figure 1). Coronal sections of 4 µm were made with a microtome of the midportion of the anterior horn, the midbody, and the posterior horn according to the International Society of Arthroscopy, Knee Surgery and Orthopaedic Sports Medicine (ISAKOS) classification.^1^

Sections were stained with standard hematoxylin and eosin and CD31. CD31 is a specific marker for endothelial cells and can be used to visualize the microvasculature of the meniscus.^27,33^ Heat-induced epitope retrieval was performed using EnVision FLEX TRS, high pH, to unmask antigens and enhance antibody binding. Subsequently, sections were incubated with the primary antibody (CD31, clone JC70A, 1:200; Dako, Agilent). To prevent nonspecific background staining, sections were incubated with peroxidase-blocking reagent for 3 minutes. Sections were incubated with a secondary antibody (mouse linker) and a labeled polymer (EnVision FLES HRP). Staining was performed using EnVision FLEX substrate working solution. Finally, sections were counterstained using Mayer’s hematoxylin solution. All sections were digitized by scanning using the Philips IntelliSite Pathology Solution (Philips Medical Systems). The sectioning and staining of all specimens were performed at the same pathology department (Haaglanden Medical Center, The Hague, The Netherlands) and checked by 1 experienced pathologist (H.M.H.).

### Microvascular Measurement

**Figure 2.**
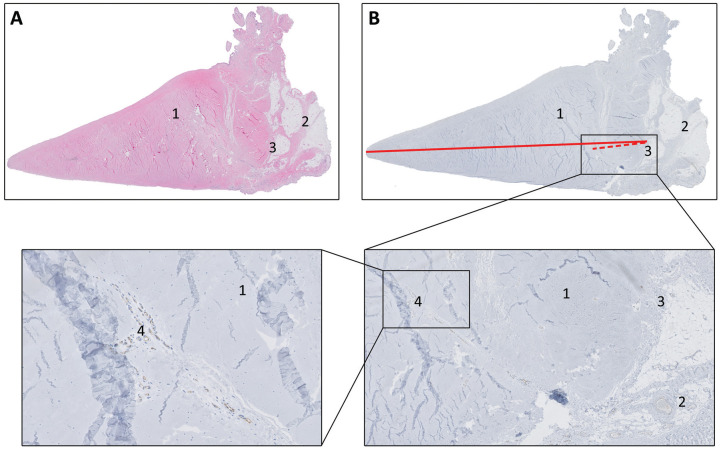
Tissue sections stained with (A) hematoxylin and eosin and (B) immunostaining (CD-31). Meniscus (*1*); capsule (*2*); meniscocapsular junction (MCJ) (*3*); blood vessel (*4*). The depth of microvascular penetration is calculated by dividing the distance between the MCJ and the most centrally located blood vessel (dotted red line) by the total width of the meniscus (solid red line) multiplied by 100%.

The extent of microvascular penetration of radial branches from the capillary plexus in the capsule into the meniscus was assessed. The depth of microvascular penetration was calculated on the digitized sections by dividing the distance from the meniscocapsular junction (MCJ) to the most centrally located blood vessel by the total width of the meniscus (Figure 2).^32,33^ The total width of the meniscus was measured as the distance between the MCJ and the most centrally located end of the meniscus.^7,33^ Measurements were performed by one researcher (T.J.N.v.d.L.) and verified by another researcher (H.M.H.), both blinded to clinical data and patient characteristics.

### Statistical Analysis

A power calculation was performed before the start of the study based on 4 primary research questions: What is the correlation between the extent of meniscal vascularization of the lateral meniscus and (1) age, (2) smoking, (3) degree of osteoarthritis, and (4) history of CVD? History of CVD included coronary heart disease, stroke or transient ischemic attack, and peripheral arterial disease. The Bonferroni correction was applied to mitigate the risk of type 1 error that arises with multiple hypothesis testing. For the present study, an alpha (α) of 0.0125 (0.05 ÷ 4) was used for the sample size calculation. To detect a correlation of 0.25 with a power of 80%, 174 lateral menisci were needed. PASS Sample Size Software was used to calculate the sample size (PASS 2020 Power Analysis and Sample Size Software). The Pearson correlation coefficient was used for continuous variables, the point-biserial correlation coefficient for dichotomous variables, and Spearman rank correlation coefficient for ordinal variables. The null hypothesis was that the correlation coefficient was zero. The statistical significance for the primary outcome measures was set at *P* < .0125 to correct for multiple testing. For the primary analyses, the degree of vascular penetration into the posterior horn of the lateral meniscus was used, as the section of this horn was available for most patients. If no adequate section of the posterior horn was available for a patient, either the midbody or anterior horn of the lateral meniscus was used, as no differences in depth of vascular penetration among the different radial locations within the lateral meniscus were expected.^7^ Vascularization between different horns within each meniscus was compared with the Skillings-Mack test, as the vascular ingrowth was nonnormally distributed and there were missing data.^6^ Means were reported with 95% CI or standard deviation for normally distributed variables. The median and interquartile range (IQR) were provided for nonnormally distributed variables.

A linear mixed-effects model (LMM) was estimated to study the association between the patient characteristics and the depth of microvascular ingrowth into the menisci adjusted for meniscal side (lateral or medial meniscus) and radial location. The LMM deals effectively with missing values and takes the within-subject correlation into account between multiple measurements within the same patients. Given the nonnormal distribution of the degree of vascular penetration, the outcome variable was log-transformed and computed as log10(Vascular ingrowth (%) + 1). The model included radial location (anterior horn, midbody, or posterior horn) and variables for the specific patient characteristics (age, smoking, degree of osteoarthritis, and history of CVD) as fixed effect. Moreover, the model included meniscal side and an interaction term between meniscal side and radial location. Patient was included as random effect and unstructured variance-covariance matrix used. Visual inspection of the residual plots suggested that there was no violation of the linearity assumption, homoscedasticity, and normality. In the LMM, the null hypothesis was that the regression coefficients for each variable were equal to zero. Analyses were performed using SPSS software Version 29.0 (IBM SPSS Statistics) and R computational software Version 4.2.1 (R Foundation).

## Results

**Figure 3.**
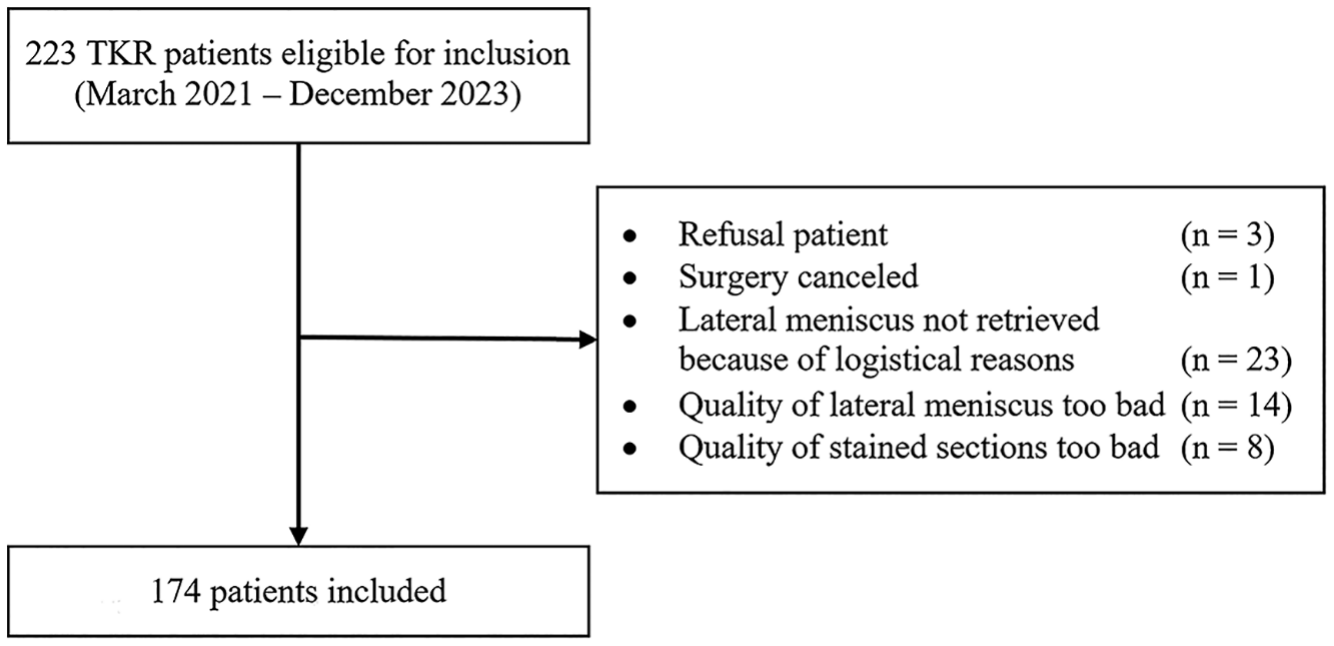
Flowchart inclusion. TKR, total knee replacement.

Of the 223 patients undergoing TKR who were eligible for inclusion, 174 were included. Three patients did not want to participate in the study and 1 patient could not be included because the surgery was canceled. A further 23 patients were excluded for logistical reasons, which included rescheduling of TKR surgery or when the operating surgeon had forgotten to collect the menisci or was informed too late about inclusion of a patient. Fourteen patients were excluded because the lateral meniscus was too fragmented macroscopically or not much was left of the meniscus. Eight patients were excluded because of poor quality of the stained sections and therefore adequate measurements were not possible. Finally, the menisci of 174 patients were included in the study (Figure 3).

Patient characteristics are presented in Table 1. The mean age of the study cohort was 73.1 years (range, 52-94 years), and 116 patients (67%) were women. The degree of osteoarthritis (Kellgren-Lawrence [KL] classification) was equally distributed among the study group. Sixteen patients (9%) smoked and 46 (26%) had a history of CVD.

**Table 1:** Patient Characteristics^*a*^

Parameter	Values
No. of patients	174
Age, y	73.1 ± 8.0
Smoking, yes	16 (9)
History of cardiovascular disease, yes^*b*^	46 (26)
Kellgren-Lawrence classification^*c*^	
0	9 (5)
I	40 (23)
II	50 (29)
III	40 (23)
IV	35 (20)
Sex	
Male	58 (33)
Female	116 (67)
Side knee	
Right	86 (49)
Left	88 (51)
ASA classification	
I	12 (7)
II	106 (61)
III	56 (32)
Body mass index, kg/m^2^	29.2 ± 4.9
Hypertension, yes	116 (67)
Diabetes mellitus, yes	31 (18)

a The values are given as the mean ± SD or as the number of patients with the percentage in parentheses. ASA, American Society of Anesthesiologists.

b Cardiovascular disease included coronary heart disease, cerebrovascular disease, and peripheral arterial disease.

c The classification was applied to the lateral side of the knee (as the degree of vascularization of the lateral meniscus was the primary outcome).

After sectioning and staining of the specimens, 145 CD-31 sections of the posterior horn of the lateral meniscus were of sufficient quality to perform the measurements. For the midbody and anterior horn of the lateral meniscus, respectively, 124 and 122 immunostained sections of sufficient quality were available. At least 1 section of the medial meniscus was available for 102 patients.

### Depth of Microvascularization

**Figure 4.**
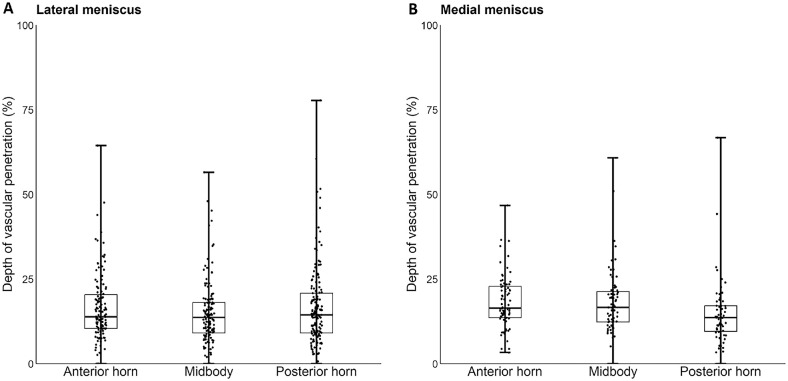
Depth of vascular penetration by radial location of the (A) lateral and (B) medial meniscus. The box represents the 25th to 75th percentiles, with the median noted by the center line inside the box. Whiskers extend from the minimum to maximum values.

The depth of vascular penetration ranged from 0% to 78% into the lateral menisci and from 0% to 67% into the medial menisci. No differences were found for the depth of vascular penetration between the anterior horn (median, 13.9%; IQR, 10.4-20.4), midbody (median, 13.7%; IQR, 9.1-18.1), and posterior horn (median, 14.4%;, IQR, 9.1-20.8) of the lateral meniscus (*P* = .220) (Figure 4). Within the medial meniscus, the posterior horn (median, 13.6%; IQR, 9.6-17.1) showed significantly less vascularization compared with the midbody (median, 16.6%; IQR, 12.3-21.3) and the anterior horn (median, 16.5%; IQR, 13.6-22.8) (*P* = .002).

### Correlations Between Patient Characteristics and Depth of Microvascularization

**Figure 5.**
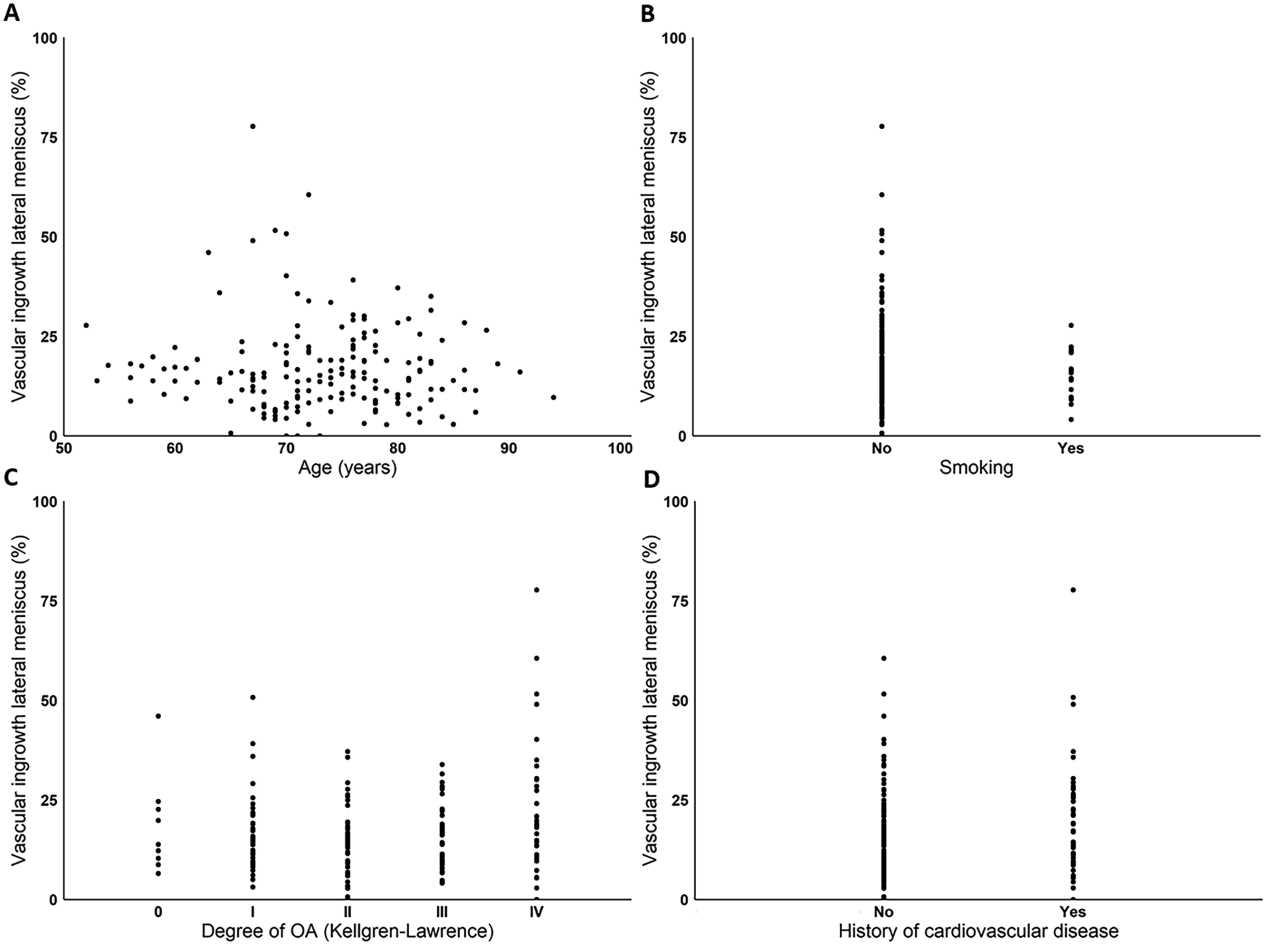
Depth of vascular penetration of the lateral meniscus by (A) age, (B) smoking status, (C) degree of osteoarthritis, and (D) history of cardiovascular disease. OA, osteoarthritis.

No significant correlations were found between depth of microvascular penetration and age (–0.049; 95% CI, –0.196 to 0.101; *P* = .523), smoking (–0.048; 95% CI, –0.196 to 0.101; *P* = .528), degree of osteoarthritis (0.109; 95% CI, –0.041 to 0.253; *P* = .153), or history of CVD (0.158; 95% CI, 0.009 to 0.296; *P* = .038) within the lateral meniscus (Figure 5). Patient characteristics such as sex (–0.083; 95% CI, –0.229 to 0.066; *P* = .273), history of diabetes mellitus (–0.017, 95% CI, –0.165 to 0.132; *P* = .826), and history of hypertension (0.054; 95% CI, –0.095 to 0.202; *P* = .472) also showed no correlation with microvascular ingrowth.

### Linear Mixed-Effects Model

The LMM analysis showed no association between the depth of vascular penetration in the meniscus and age (*P* = .534), smoking (*P* = .925), degree of osteoarthritis (KL grade I, *P* = .911; KL grade II, *P* = .617; KL grade III, *P* = .870; KL grade IV, *P* = .489), or history of CVD (*P* = .084), adjusted for meniscal side and radial location.

## Discussion

This study found no significant associations between depth of microvascular penetration into the meniscus and age, smoking, degree of osteoarthritis, or history of CVD. Considerable variation in the degree of meniscal vascularization of both the lateral and medial meniscus was found in a large group of patients.

Arnoczky and Warren^2^ published their landmark paper on the microvascularization of the meniscus in 1982, in which the medial and lateral menisci of 20 ink-injected cadaveric specimens (aged 53-94 years) were studied. The investigators found that the degree of vascular penetration varied among specimens, ranging from 10% to 25% of the meniscal width in the lateral meniscus and from 10% to 30% in the medial meniscus. Our study showed that the range of vascular penetration into both the medial and lateral meniscus is much greater. Furthermore, although Arnoczky and Warren^2^ did not provide any statistical explanation, they reported that there was no difference in the microvascular anatomy of the meniscus with regard to age, sex, or race. In our large cohort of patients, we showed that there was no pattern of vascular regression with increasing age or different sex.

Within the lateral meniscus, no differences were found in the depth of vascular penetration between the different radial locations. In contrast, the posterior horn of the medial meniscus showed significantly less vascularization compared with the anterior horn and midbody of the medial meniscus. These findings are in accordance with those of Crawford et al,^7^ who studied the depth of vascular penetration into the menisci of 13 cadaveric knees (aged 22-34 yeas). Crawford et al reported no significant differences among the 3 radial locations within the lateral meniscus but observed significant variation in the degree of vascular penetration into the medial meniscus, with the posterior horn having a significantly smaller depth of penetration. Median values of all radial locations in our study were comparable with those in a younger population as reported by Crawford et al, which may suggest that our findings regarding the absence of associations between patient characteristics and microvascular penetration could be generalized to the complete adult population.

Although age is often considered a contraindication for meniscal repair, recent studies have shown that age of 40 years or older is not associated with an increased failure risk of meniscal repair.^16,26^ Our results show that the depth of vascular penetration is not a valid argument against meniscal repair in older adults, as age was not associated with the depth of microvascularization. In contrast, Michel et al^22^ included menisci from 28 patients aged between 3 and 79 years and reported a negative linear trend with increasing age and microvascular density of the meniscus. The latter study only included 11 patients older than 50 years. As our study did not include patients younger than 50 years, some caution is required to extrapolate our findings to the complete adult population. Importantly, whereas Michel et al assessed the vascular density of the meniscus, we studied the depth of microvascular penetration. Therefore, care must be taken in the interpretation of our results. On the basis of our data, we can conclude that there is no association between age and depth of microvascularization, but we cannot conclude that there is no association between age and microvascular density.

Besides the vascular status of a meniscal tear, other factors that influence the healing rate of a tear after repair have been described.^8,30^ As for the degenerative knee, conflicting literature exists. Whereas Ashraf et al^3^ reported that tibiofemoral chondroplasty was associated with increased vascular penetration along the midline of the meniscus, Danzig et al^9^ reported no difference in blood supply within degenerative menisci compared with normal menisci and Wang et al^34^ even reported fewer blood vessels in the vascular region of degenerative menisci. Our study showed no association between the depth of vascular penetration and degree of osteoarthritis. To the best of our knowledge, no previous study has investigated the effect of smoking on meniscal vascularization, yet smoking has been identified as a risk factor for failure of meniscal repair.^4,31^ However, the dose-dependent effects of smoking on the healing capacity of the fibrocartilaginous tissue of the meniscus are still unknown.^35^ We did not observe decreased vascular penetration into the meniscus in actively smoking patients compared with nonsmokers. Although we did not study the influence of actual pack-years of smoking on meniscal vascularization, limited vascular meniscal supply may not be the cause of increased failure rates of meniscal repair in smokers. Finally, although we hypothesized that patients with a history of CVD would show decreased microvascular penetration, this was not observed.

A strength of this study is its large sample size. This was the first study with adequate sample size to assess associations between patient characteristics and meniscal vascularization. Another strength was the use of immunohistochemistry for the assessment of blood vessels. Previous anatomic studies on meniscal vascularization used injection techniques, which only visualizes vessels that are filled with ink, and the absence of vessels in dense connective tissue may be caused simply by insufficiently filled vessels.^25,29^ Our findings may guide future research on meniscal microvascularization. As no associations were found, it may be impossible to estimate the degree of vascularization of the meniscus based on demographic data of individual patients. The latter may stimulate the development of (intraoperative) imaging techniques for the objective assessment of meniscal microvascularization. For example, using indocyanine green fluorescence-guided knee arthroscopy could potentially contribute to investigating patient-specific meniscal vascularization during surgery.^17,33^

Some limitations should be noted. Some menisci of poor quality were excluded (macroscopically or after sectioning), so no adequate immunohistological staining and subsequent measurements could be performed. These excluded menisci could have had different microvascular patterns, potentially influencing our results. Nonetheless, we included a considerable number of patients with severe osteoarthritis, and therefore it was still possible to assess the association between degree of osteoarthritis and meniscal microvascularization. In the primary analysis of this article, we assess the correlation between KL grade of the lateral compartment of the knee and depth of vascular penetration into the lateral meniscus. However, a higher degree of osteoarthritis in the medial compartment may potentially also affect the vascularization of the lateral meniscus. Also, young active patients (aged <50) with acute traumatic meniscal tears are usually offered arthroscopic surgery to repair or (partially) resect the meniscus. We included only the menisci of patients older than 50 years, so associations between patient characteristics and degree of vascularization may differ in the younger adult population. However, performing a comparable study in young adults with similar statistical power as the present study is complex because of the limited availability of complete meniscal specimens of young adults.

## Conclusion

There is a wide range in the degree of microvascular penetration into the meniscus among adults older than 50 years. However, the depth of microvascularization was not associated with age, smoking, degree of osteoarthritis, or history of CVD.
